# The risks behind the widespread use of siliconized syringes in the healthcare practice

**DOI:** 10.1186/s40942-021-00338-0

**Published:** 2021-10-30

**Authors:** Gustavo Barreto Melo, Yehuda Shoenfeld, Eduardo Büchele Rodrigues

**Affiliations:** 1 Department of Ophthalmology, Federal University of São Paulo/Paulista School of Medicine, São Paulo, Brazil; 2 Hospital de Olhos de Sergipe, Rua Campo Do Brito, 995, Aracaju, Brazil; 3 Zabludowicz Center for Autoimmune Diseases, Sheba Medical Center, Tel-Aviv University School of Medicine, Tel-Aviv, Israel; 4 Department of Ophthalmology, SSM Health Saint Louis University Hospital, Saint Louis University, Saint Louis, USA

**Keywords:** ASIA syndrome, Autoimmune disease, Breast implant, Dermal filler, Inflammation, Intravitreal injection, Silicone oil, Subcutaneous injection, Syringe, Vaccination

## Abstract

Injections are widely performed in the healthcare practice. Silicone has long been thought to be an inert and harmless material. Although used for decades in medical implants, including heart valves, breast implants, and as a tamponade for retinal detachment surgery, silicone oil might have deleterious effects. Agitation of the syringe to expel air at the moment of drug preparation not only leads to silicone oil release but also to therapeutic protein aggregation. Lab studies have shown that silicone oil microdroplets can act as an adjuvant to promote a break in immunological tolerance and induce antibody response. Similarly, recent studies have suggested a causal link between agitation of siliconized syringes and ocular inflammation after intravitreal injection. Systemically, silicone oil has been reported in association with autoimmune diseases and skin granuloma after either direct injection of dermal fillers or secondary leakage from silicone breast implant. However, it has not been established yet a potential link between the silicone oil released by the syringes and such relevant systemic adverse events. Few professionals are aware that agitation of a siliconized syringe might lead to silicone oil release, which, in turn, acts an adjuvant to an increased immunogenicity. We strongly recommend that every healthcare professional be aware of the use of silicone oil in the syringe manufacturing process, the factors that promote its release and the potential complications to the organism. Ultimately, we recommend that safer syringes be widely available.

## Background

**Fig. 1:**
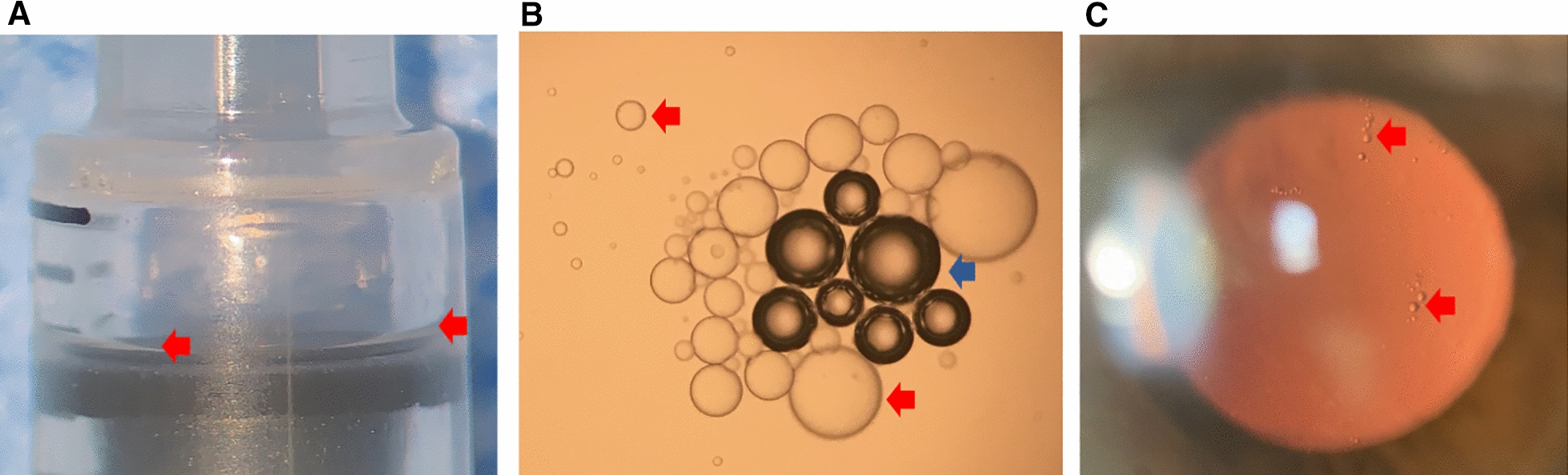
Illustrative images of silicone oil from the syringe. **A** Silicone oil layer (red arrowhead) in the barrel of the syringe adjacent to the rubber stopper. **B** Multiple silicone oil droplets (red arrowhead) seen at light microscopy released from the syringe. Note the presence of air bubbles (blue arrowhead). **C** Silicone oil droplets (red arrowhead) in the anterior vitreous of a patient who had recently recovered from non-infectious endophthalmitis following intravitreal injection of an antiangiogenic drug

Injections are widely performed in the healthcare practice. Most of them are siliconized in order to allow for a better gliding of the piston (Fig. 1). Silicone oil has been long thought to be an inert and harmless material, and consequently it has been used for decades in medical implants including heart valves, breast implants, and as a tamponade for retinal detachment surgery [1–3]. Unfortunately, silicone oil might have deleterious effects.

## Main text

Our research group has deeply studied the complications of the syringe, silicone oil, and injection technique in ophthalmology [1]. From a clinical observation of noninfectious endophthalmitis associated with the syringe, we developed lab studies to assess silicone oil qualitatively and quantitatively. We found that most commercially available syringes are coated with silicone oil, and the oil is especially released by agitation by flicking/tapping [1]. Kim et al. have recently published a paper that supports these findings [4]. In addition to the lab-based research, we reported on the preliminary data of a clinical trial assessing whether agitation of a siliconized syringe could lead to the detection of inflammatory cells in the anterior chamber of the eye following an intravitreal injection of an antiangiogenic fusion protein [1]. We found a remarkable increase in the rate of these cells when the syringe was agitated in comparison to the no-agitation group.

Agitation of the syringe to expel air at the moment of drug preparation or even during transportation, in prefilled syringes, not only leads to silicone oil release but also to therapeutic protein aggregation, which might ultimately lead to increased immunogenicity [5]. Lab studies have shown that silicone oil microdroplets can act as an adjuvant to promote a break in immunological tolerance and induce antibody response [5]. An increased secretion of several innate cytokines from human peripheral blood mononuclear cells and also in the plasma concentrations of antidrug antibodies was found when a siliconized syringe was agitated in comparison to both an unagitated counterpart and to an agitated silicone oil-free syringe [5].

Myodesopsia is another complication of the release of silicone oil into the eye. The complaint of floaters of different sizes and shapes is commonly bothersome, leading some individuals to require vitrectomy for their treatment. These are usually due to vitreous condensations or detachment throughout life. Silicone oil droplets, in their turn, are known to cause these symptoms. Although the minority of patients end up complaining about this matter, it has been recently acknowledged that the prevalence of this problem is much higher than previously anticipated [1]. Additionally, a survey carried out by the American Society of Retina Specialists has shown that about 5% of their US members have done vitrectomy to treat patients with symptomatic floaters while 2% have seen patients seeking legal action [6]. If we consider that at least 25 million intraocular injections are performed yearly worldwide, awareness of this association becomes even more important.

Besides its likely role as an adjuvant to ocular inflammation and myodesopsia, silicone oil has been reported in association with autoimmune diseases, systemic embolism and sclerotic lipogranuloma [2, 3, 7, 8]. A clinical entity resulting from breast silicone implants has long been acknowledged. Recently, it has been suggested that the silicone implant incompatibility syndrome might be part of the spectrum of the autoimmune/inflammatory syndrome induced by adjuvants (ASIA syndrome) [2, 3]. Autoimmune reactivity develops, with subsequent symptoms including myalgias, arthralgias, chronic fatigue, sleep disturbance, and cognitive impairment [2, 3]. The etiology of this pathology is supposed to be secondary to chronic leakage of silicone oil, which is transported to the lymphatic by phagocytosis. Subsequently, it may cause adenopathy, with the potential for systemic immune hyperreactive response [3].

Subcutaneous injection of silicone oil in order to smooth out for esthetical reasons has become very popular, especially in those groups highly focused on their physical image. The injected subcutaneous silicone seems to migrate from the interstitial subcutaneous tissue into the general blood stream, resulting in a potentially fatal systemic silicone embolism [9]. Additionally, local sclerotic lipogranulomas might occur if injected in the fat tissue [7]. Another complication of dermal fillers includes pneumonitis, leading to respiratory failure [10].

Considering the aforementioned information, we believe that: (1) all products with the potential to release particles into the eye should be tested; (2) silicone oil-free and low particle-siliconized syringes should be developed and tested; (3) experimental models that better allow for assessing the impact of those particles into the eye should be developed; and (4) more clinical studies that better characterize the extent and clinical relevance of the silicone oil droplets released by the syringes should be carried out.

## Conclusions

These data are widely applicable to the whole healthcare practice. Few professionals are aware that agitation of a siliconized syringe might lead to silicone oil release, which, in turn, acts an adjuvant to an increased immunogenicity. Additionally, no large clinical research has ever studied the potential link between siliconized syringes, agitation and systemic disorders. Ultimately, we recommend that safer syringes be widely available, and that no agitation be performed by those professionals doing injections.

## Data Availability

Not applicable.

## Abbreviation

ASIA syndrome: Autoimmune/inflammatory syndrome induced by adjuvants
